# Predicting properties of antifungal drug molecules using neighborhood degree topological indices

**DOI:** 10.1038/s41598-026-61020-9

**Published:** 2026-07-07

**Authors:** Abdullah Ahmed Almulla, Ibrahim Irfan, Zeeshan Saleem Mufti, Mazen Omar Almulla, Gamachu Adugna Ganati

**Affiliations:** 1 Department of Special Education, College of Education, King Faisal University, Al-Ahsa, 31982 Saudi Arabia; 2 Department of Mathematics and Statistics, The University of Lahore, Lahore, Pakistan; 3 Department of Education and Psychology, College of Education, King Faisal University, Al-Ahsa, 31982 Saudi Arabia; 4 Department of Mathematics, Wallaga University, Nekemte, Ethiopia

**Keywords:** Neighborhood degree of vertex, Antifungal drugs, Topological index, Chemical graphs, Drug molecules, Regression models, Chemistry, Mathematics and computing

## Abstract

Topological indices are an important part of chemical graph theory as a representation of the molecular structure and a predictor of the physicochemical properties. In this paper, nine neighborhood degree sum based topological indices are use as indicators of predictive potential in a QSPR analysis of twenty antifungal drug molecules. Linear, quadratic, and cubic regression models were used to test the relationships between the structural properties and important physicochemical properties, such as boiling point, density, enthalpy of vaporization, flash point, refractive index, molar refractivity, polarizability, surface tension, and molar volume. The findings also revealed that a cubic regression model provided the most optimal overall performance with the highest level of predictability in the form of molar refractivity and polarizability being the neighborhood inverse product index $(ND_{4} )$ ($R^{2}$ = 0.98). These results assert that neighborhood based topological descriptors are effective, especially in modeling the refractivity related and electronic properties of drug molecules.

## Introduction

In any system where entities are mutually interconnected, the solution to a particular puzzle gives rise to a conceptual field that enables the visual interpretation of relations. Graph theory offers a formal framework for representing and analyzing such problems by modeling them as sets of vertices connected by edges. Researchers have applied graph theoretic concepts to address problems in disciplines such as physics and chemistry, as well as to solve various logical puzzles. Furthermore, graph theory provides tools that enhance theoretical understanding and serve as a basis for modeling complex phenomena^1^. Although graph theory underpins much of modern technology, this practical role often reveals only a limited perspective on its full scope. Its significance is clearly observable in the architecture of social media platforms, internet infrastructures, and communication networks^2^, as well as in emerging fields such as artificial intelligence, where graph based models form a core component of algorithmic design and knowledge representation.

The integration of graph theory with other scientific disciplines has facilitated the emergence of multiple new research fields. One such field is mathematical chemistry, which offers a framework for addressing chemical problems through mathematical methods. Researchers employ tools including graph theory and group theory to persue chemical compounds and reactions. These tools are utilized not only for analysis and modeling but also for the discovery and prediction of novel compounds and their associated properties. Within mathematical chemistry, a subfield termed as chemical graph theory further advances understanding by representing atoms as vertices and chemical bonds as edges, thus improving the understanding of chemical behavior^3^. The graphical representation of molecular structures has led to the formulation of specialized numerical quantities named as topological indices. These indices serve to encode structural patterns within chemical compounds. The concept of topological indices gained prominence following after Harold Wiener’s 1947 study, in which he successfully modeled the boiling points of Paraffin hydrocarbons. This work introduced the first topological index, subsequently referred as the Wiener index^4^. Wiener’s contribution established a methodology for constructing numerical correlations between the physicochemical properties of a molecule and its graph based structural representation. Following the introduction of the Wiener index, mathematicians developed numerous other descriptors. Among these is the Szeged index^5^, which constitutes a refinement of the Wiener index and is specifically formulated for application to cyclic molecular graphs. Subsequently, researchers proposed various topological indices based on distinct graph theoretical concepts, including vertex degree, topological distance, and neighborhood properties. Within this classification, degree based topological indices have gained substantial attention from mathematicians due to their computational efficiency and strong predictive capacity. These indices typically capture molecular structural features such as branching patterns and connectivity. Representative examples include the Sanskruti index^6^ and the Zagreb index, as well as improved variants such as modified reverse degree type indices, which exhibit sensitivity to diverse molecular structural characteristics. Despite their simplicity, degree based descriptors demonstrate applicability across a wide range of physicochemical property predictions, thereby reinforcing their scientific relevance. With the passage of time, development in QSPR and significance of Zagreb lead toward the invention of enhanced foam of it. Following this trajectory, Çiftçi (2025) used Zagreb Rho indices to predict the physicochemical properties od Benzene and their derivatives and find strong predictive power for various properties^7^. In same pattern, Çiftçi (2025) examine Benzene and their derivatives in different manner by using Zagreb Eta^8^ which gives high $R^{2}$ value for polarizabilty and molar refractivity. Expanding the work on same compounds, Çiftçi (2025) study on other novel set of Zagreb family named as Zagreb upsilon^9^ which target pi electron energy of investigated compounds. Çiftçi (2025) introduced the Zagreb Omicron index^10^, while Yamaç employed the Zagreb Tau indices^11^ to model the physicochemical behavior of benzene and its derivatives, reporting strong predictive capability for properties including *π* electron energy, molar refractivity, and polarizability. Considering the potential of degree based indices Mondal and Das used exponential version of degree based index named as exponential augmented Zagreb^12,13^. Subsequently, Das also study different benzenoid hydrocarbons and octane isomers with the help of exponential geometric arthmatic index^14^.

Chemicals have become an integral component of our daily lives and are found in many different forms and structures. Similarly, as living organisms, we encounter various diseases, and sometimes we neglect to provide our bodies with essential nutrients such as vitamins and proteins. Keeping these problems in mind, solutions emerge in the form of pharmaceutical drugs. A drug supplies the body’s necessary nutrients, prevents infection by disease causing microorganisms, and enhances immune function. In recent years, plant based extracts have served as alternatives to synthetic drugs however they largely been replaced by chemical compounds composed of various elements. Over time, new diseases spread and new pandemic emerge, increasing the importance of the pharmaceutical industry in addressing such challenges. In addition, pharmacists face the issue of time consuming experimental procedures, which delays drug development and consequently leads to numerous deaths. To accelerate development and improve drug quality, topological indices and QSPR analysis are required. This approach simplifies and advances the understanding of chemistry. By applying a topological index, we reduce a molecular graph to a single numerical value. This value is subsequently employed in QSPR analysis to establish a quantitative link between molecular structure and its corresponding properties^15–17^. In the present investigation, we selected twenty antifungal compounds: marbofloxacin, 1-octacosanol, l-threonic acid, menthyl salicylate, florfenicol, emylcamate, fluindione, levalbuterol, arbutin, amoxate, fluconazole, terbinafine, clotrimazole, miconazole, voriconazole, tavaborole, butenafine, econazole, oxiconazole, and sertaconazole. Our goal is to determine which index best explains the physicochemical behavior of each drug.

The principal innovation of this study is close study of open neighborhood based topological indices of drug molecular graphs. Degree based indices provide information on the number of edges owned by a vertex. In contrast, the selected indices use the concept of the neighborhood degree, which provides specific information about the surrounding atoms tied to an atom. This provides superior insight into the molecular structure. Consequently, these indices indicate better performance in QSPR modeling. We also found that certain indices, particularly $ND_4$, yielded very high $R^2$ values in cubic regression models, implying that they are powerful predictors of the significant physicochemical attributes of drugs.

However, this study has some limitations. As we are studying a limited dataset comprising only 20 antifungal drugs, using higher order regression models, such as quadratic and cubic, may lead to overfitting because higher order regression models are sensitive to small fluctuations in data. Also these models are flexible and contain more parameters (like quadratic has 3 parameters and cubic has 4 parameters), which results in a high $R^2$ value, but predictive power for unseen data may end up to different results and values.

A cell is the fundamental unit of a living organism that is responsible for performing different activities in the body. Disturbances in their work lead to different disorders, one of which is related to the human neurological system and is called Parkinson’s disease, which is caused when problems arise in dopamine production cells. Medication is the primary approach for recovery from this disease, and with the help of QSPR, it becomes easy to treat^19^. The environment plays an important role in determining how healthy you live. The environment is a sign of good health; in the same way, it also causes the development of cancer in the body. The lungs are an important organ for breathing, but a bad environment and tobacco use can affect the lungs with cancer, which slowly invades the whole lung and its surrounding tissues. Drug therapy helps to recover damaged tissues, and with the help of QSPR and other tools, we can improve the quality of medicines^20^. To understand the biological processes or known life on Earth, we must understand a class of chemicals known as organic compounds, which are present in everything essential for life. A combination of hydrogen, carbon, and other elements makes proteins and carbohydrates, like organic compounds in the same way they make a class of hydrocarbons that are harmful to humans, named polycyclic aromatic hydrocarbons (PAHs)^21^. Before corona there is disease with most death rate in record and it is caused by bacillus Mycobacterium tuberculosis named bacteria which direct target your lungs and also invade to your other organs like kidney or liver. Most sufferers are adults, and without proper medication, the disease can lead to death. According to a WHO report, 50 percent of adults die from TB. The combination of chemistry with graph theory has put the process of drug manufacturing on a fast track, and QSPR has made things easy^22^.A complex network of airways and other passages spread throughout the human body, which is vital for the exchange of oxygen and carbon dioxide from the lungs. Narrowing or swelling of these airways not only damages the lungs but also affects the entire human respiratory system. This condition is called asthma, which is incurable but manageable. Medication in form of inhaler relief pain of it and also by understanding and managing trigger which cause problem. QSPR and topological indices where split and convert molecule into number and give relief us from wet lab testing type expensive experiments too^23^

Your body has a natural system to defend you from viruses, bacteria, and other disease-causing germs, called the immune system. Some special cells, called CD4, are part of the immune system, which finds germs and alerts other defending cells that are destroyed by the HIV virus, which not only weakens your defence system but also destroys it. HIV can be transmitted through various mediums, such as used needles, body fluids, and from mother to child during pregnancy^24^. As humans, it matters how we interact with our surroundings and with other humans. The brain plays a major role in deciding how we behave and relate to others. However, due to factors such as addiction, depression, or anxiety, brain functionality is affected, which slowly takes the shape of mental disorders. Such disorders not only disturb and negatively impact social life but sometimes also affect our way of thinking. Different addictions and depression make a large group of the population suffer of it. To address this situation, we established the foundation of an experimental technique that includes QSPR, which simplifies the treatment of such disorders^25^. But to examine and study selected antifungal drugs from various view points, we chose neighborhood topological indices. These indices primarily target solving problems related to molecular connectivity and chemical bonding, along with the properties arising from them. Connectivity and bonding are fundamental characteristics of a chemical compound because they allow prediction of other major properties, such as solubility and boiling point. These properties are essential for a range of products, including pharmaceutical drugs and petroleum derived substances.

## Preliminaries

### Definition 1

Graph theory provides the concept of graph through which we can demonstrate the relationship between objects. It consists of two sets: one is vertex set and second one is edge set.

### Definition 2

The order of the graph indicates the number of vertices in the graph. This is represented by O(G).

### Definition 3

The size of the graph indicates the number of edges in the graph. This is represented by E(G).

### Definition 4

The neighborhood is calculated for each vertex in such a way that if G is a graph and u is a vertex of it, then the neighborhood of u is simply composed of a set that contains all vertices that share edges with the u vertex, and it is written as N(u). Mathematically, we explain it as

1 $$\begin{aligned} N(u) = \{ v \in V(G) | uv \in E(G)\} \end{aligned}$$

Neighbourhood topological indices which we use in our paper are displayed in Table 1

**Table 1:** Comparative edge distribution of different drugs.

Index names	Symbol	Mathematical formula
First neighbourhood Zagreb^26^	$NM_1$	$NM_1 = \sum _{v \in V(G)} \delta (v)^2$
Second Neighbourhood Zagreb^26^	$NM_2$	$NM_2 = \sum _{uv \in E(G)} \delta (u)\delta (v)$
Neighbourhood atom bond connectivity^27^	NABC	$NABC = \sum _{uv \in E(G)} \sqrt{\frac{\delta (u) + \delta (v) - 2}{\delta (u)\delta (v)}}$
Neighbourhood forgotten	$NF_1$	$NF_1 = \sum _{v \in V(G)} \delta (v)^3$
First neighbourhood degree product^27^	$ND_1$	$ND_1=\sum _{uv \in E(G)} \sqrt{(\delta (u)\delta (v)}$
Second neighbourhood degree product^27^	$ND_2$	$ND_2 = \sum _{uv \in E(G)} \frac{1}{\sqrt{\delta (u)+\delta (v)}}$
Third neighbourhood degree product^27^	$ND_3$	$ND_3= \sum _{uv \in E(G)} \delta (u)\delta (v)[\delta (u) + \delta (v)]$
Fourth neighbourhood degree product^27^	$ND_4$	$ND_4 = \sum _{uv \in E(G)} \frac{1}{\sqrt{\delta (u)\delta (v)}}$
Fifth neighbourhood degree product	$ND_5$	$ND_5 = \sum _{uv \in E(G)} [\frac{\delta (u)}{\delta (v)}+\frac{\delta (v)}{\delta (u)}]$

In this context, the symbol *δ* denotes the cumulative sum of the degrees assigned to each vertex that lies in the neighborhood of v within the graph G where definition of neighbourhood of any vertex is explain in Eq. (1).

## Methodology and analysis

**Fig. 1:**
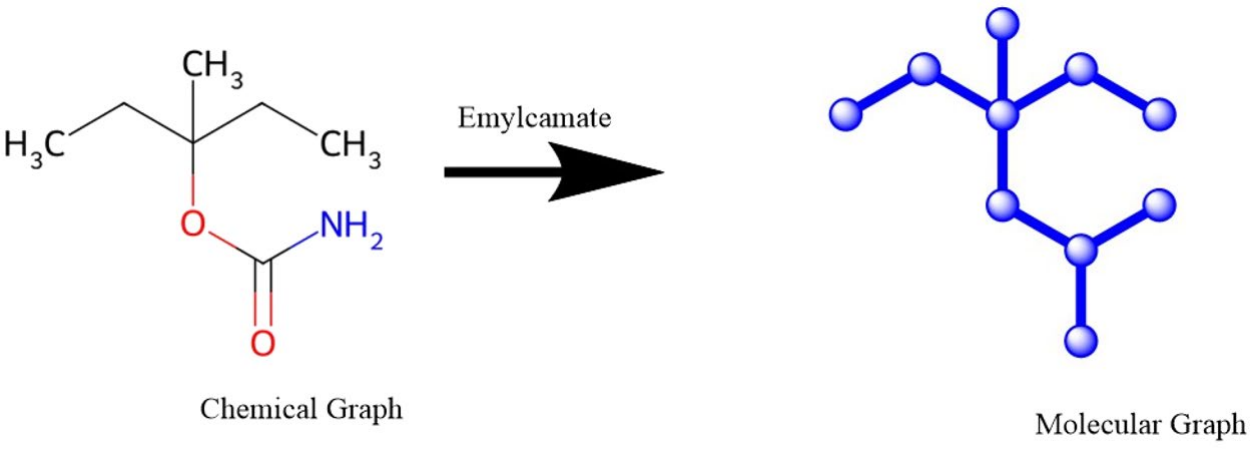
Conversion of chemical structure into molecular graph.

To extract quantitative data from the selected drugs, we first converted each chemical structure into a molecular graph and then applied the chosen topological indices. The chemical structure of emylcamate, shown in Fig. 1, serves as an illustrative example. The initial step consists of identifying the total number of atoms and bonds present in the drug molecule, as this information is essential for constructing its molecular graph. During the conversion from chemical structure to molecular graph, each non hydrogen atom treated as a vertex, following the standard convention of omitting hydrogen atoms from such representations. The bonds connecting atoms were treated as edges. In the same manner, double and triple bonds were also drawn as single lines in the molecular graph. Upon completing this conversion, we obtained an undirected connected graph, which allowed us to apply graph theoretic principles. After the molecular graph was constructed, we calculated the neighborhood degree for each vertex by summing the degrees of all adjacent vertices. Once all neighborhood degrees were determined, we applied the formulas of the selected topological indices to derive the final numerical outcomes.

**Fig. 2:**
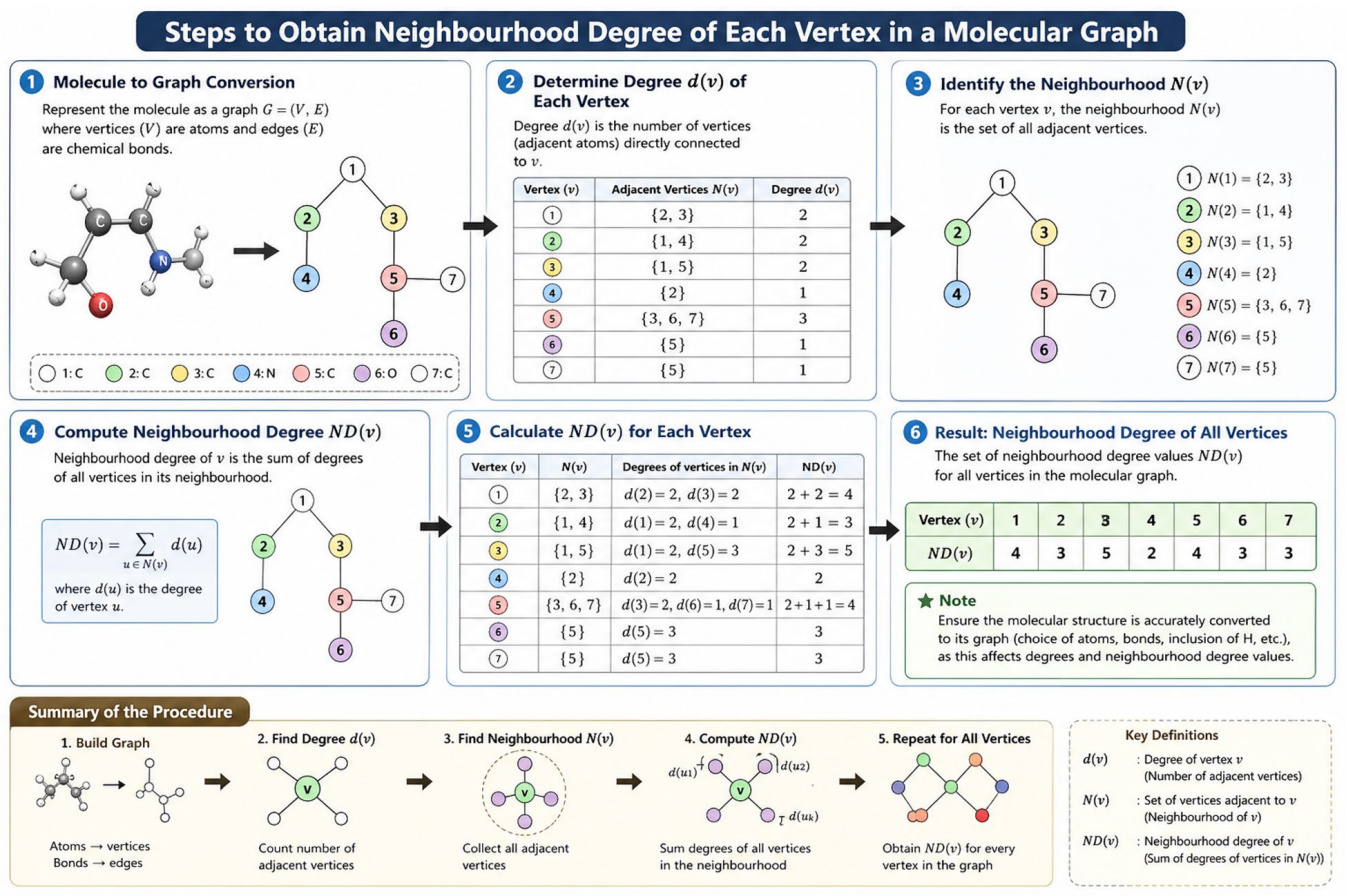
Method of finding neighbourhood degree of vertex.

After the successful conversion of the chemical structure into a molecular graph, the second step is to calculate the neighborhood degree of each vertex. However, first, we calculate the degree of each single vertex by finding how many edges a vertex owns in the graph. The degree of the vertex is denoted by d(v), where v is the vertex of the graph. The next step is to calculate the neighborhood of each vertex, which is a set of vertices adjacent to the vertex. The neighborhood of vertex is denoted by N(v), where v is the vertex. The last step is the calculation of the neighborhood degree of vertices by simply adding the degrees of adjacent vertices. The neighborhood degree is denoted by *δ (v)* where v is the vertex of the graph. A visual representation of finding neighbourhood degree of any vertex is shown in Fig. 2:

This step yields a graph upon which we apply various graph theory concepts to extract structural information encoded within the drug. To recover the structural properties, we employed the concept of edge distribution. This approach not only streamlines our calculations but also aids in understanding how atoms interact with their neighbors. It further provides a powerful tool for organizing and predicting molecular properties by targeting molecular symmetries. Below, Table 2, we present and explain how we distributed the edges of the twenty drugs under investigation which facilitating the systematic comparison.

The topological index serves as the backbone of this study because it converts a chemical compound into a single numerical value. Different types of topological indices exist, allowing us to examine drugs from various perspectives.

**Fig. 3:**
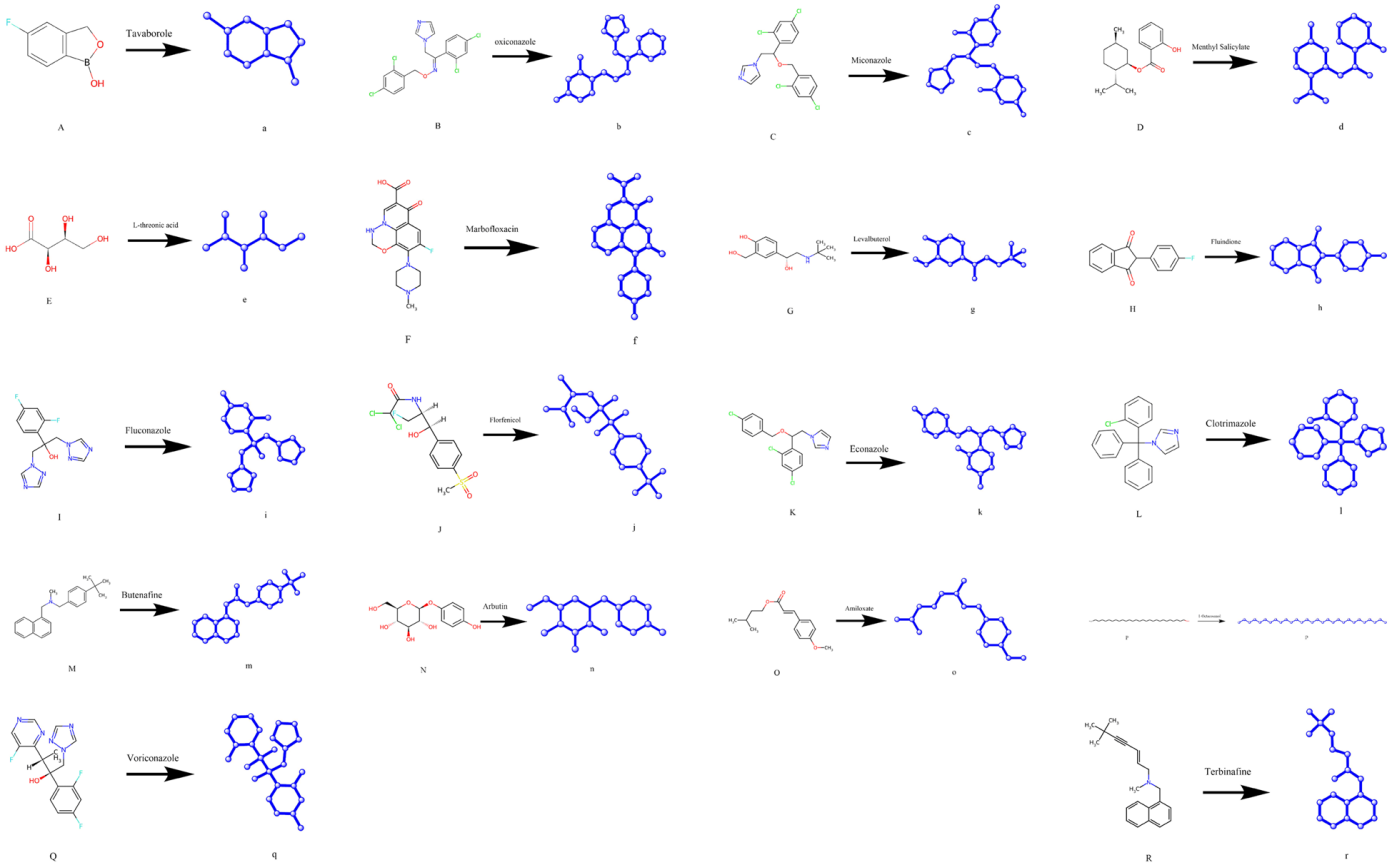
Capital letters (A–S) denote the chemical structures of the drugs, whereas lowercase letters (a–s) denote their corresponding molecular graphs.

Accordingly, in this study we selected nine open neighborhood based topological indices named as neighborhood first Zagreb ($NM_1$), neighborhood second Zagreb ($NM_2$), neighborhood forgotten index ($NF_1$), neighborhood atom bond connectivity index (NABC), neighborhood degree product ($ND_1$), neighborhood sum connectivity ($ND_2$), neighborhood mixed term index ($ND_3$), neighborhood inverse product index ($ND_4$) and neighborhood symmetric ratio ($ND_5$) whose value are displayed in Table 4 to predict and to study following drugs named as marbofloxacin, 1-octacosanol, l-threonic acid, menthyl salicylate, florfenicol, emylcamate, fluindione, levalbuterol, arbutin, amoxate, fluconazole, terbinafine, clotrimazole, miconazole, voriconazole, tavaborole, butenafine, econazole, oxiconazole and sertaconazole whose properties are shown in Table 3. All chemical and molecular graphs are shown in Fig. 3.

**Table 2:** Combined edge partitions of drugs by neighbourhood degree.

Drug	Edge Partition (degree pair : count)
Emylcamate	(2,5):2; (3,4):2; (4,7):2; (5,7):2; (7,7):1
Arbutin	(2,4):1; (4,7):1; (6,7):3; (7,7):4; (3,7):3; (5,6):2; (5,5):4; (3,5):1; (6,6):1
1-Octacosanol	(2,3):2; (3,4):2; (4,4):24
Fluindione	(5,8):2; (5,4):2; (4,4):1; (8,8):1; (8,7):2; (7,9):3; (7,3):2; (7,5):2; (5,5):4; (5,3):1
Marbofloxacin	(1,5):1; (5,5):4; (8,9):3; (6,6):1; (3,6):1; (3,5):2; (6,7):1; (3,7):1; (5,7):3; (7,9):2; (6,8):2; (6,9):1; (5,8):2; (4,5):2; (7,8):2
L-threonic acid	(3,5):2; (5,7):1; (3,7):1; (6,7):1; (3,6):1; (4,6):1; (2,4):1
Levalbuterol	(2,4):1; (4,7):1; (6,7):4; (5,7):1; (3,6):2; (5,6):4; (5,5):1; (4,5):3
Amiloxate	(3,4):2; (4,5):3; (5,5):5; (3,5):1; (5,6):5; (4,6):1; (2,4):1
Menthyl Salicylate	(3,5):3; (5,8):3; (7,8):1; (5,5):2; (5,6):2; (6,7):2; (6,6):1 ; (6,3):2 ; (6,8):2 ; (5,4):2 (4,4):1
Fluconazole	(4,4):2; (4,5):4; (5,6):5; (6,7):2; (7,8):2; (4,8):1; (8,9):1; (3,6):1; (6,9):1; (9,5):1; (5,5):2; (3,5):1; (6,6):1
Terbinafine	(4,5):7; (4,4):2; (5,7):3; (5,8):1; (8,7):2; (7,6):1; (6,5):2; (3,5):1; (5,5):1; (4,6):1
Clotrimazole	(4,4):6; (4,5):8; (5,8):6; (8,12):3; (9,12):1; (9,5):1; (5,6):1; (9,6):1; (3,6):1
Miconazole	(3,5):2; (6,5):4; (6,6):3; (5,5):5; (5,7):4; (6,7):3; (6,3):2; (4,4):1; (4,5):2; (7,7):1
Voriconazole	(3,5):1; (5,5):2; (5,9):2; (6,9):2; (6,6):1; (6,5):4; (3,6):2; (9,10):1; (4,10):1; (7,10):1; (7,6):1; (4,5):4; (4,4):2; (4,9):2; (10,9):1; (9,9):1
Tavaborole	(3,5):1; (5,5):3; (5,6):2; (6,7):1; (7,8):1; (5,8):1; (5,7):1; (6,8):1; (3,6):1
Butenafine	(4,5):4; (4,4):1; (5,8):3; (7,8):2; (7,5):3; (7,6):1; (5,6):4; (3,5):1; (6,6):1; (5,5):2; (6,8):1; (4,6):3
Econazole	(3,5):2; (5,5):7; (6,5):6; (6,6):2; (3,6):1; (6,8):1; (5,8):1; (7,8):1; (5,7):1; (6,7):1; (5,4):2; (4,4):1
Oxiconazole	(3,5):2; (5,5):4; (7,5):3; (7,6):2; (3,6):2; (6,6):3; (6,5):4; (4,5):4; (4,4):1; (7,8):1; (6,8):1; (5,8):1
Sertaconazole	(3,5):1; (5,6):2; (6,6):2; (6,8):2; (8,5):3; (5,5):4; (3,6):2; (7,8):2; (7,6):1; (6,5):2; (4,5):4; (4,4):1; (7,5):3; (8,8):1
Florfenicol	(4,6):3; (8,6):1; (8,5):2; (5,5):2; (7,5):2; (7,7):2; (3,7):1; (4,7):1; (2,4):1; (7,6):1; (6,6):1; (3,6):1; (6,5):1; (3,5):2

### Physico chemical properties of drugs

There is large spectrum of properties including physical and chemical properties through which we can represent a chemical compound. But in this investigation, we give priority to ten critical and key properties named as boiling point, density,enthalpy of vaperization, flash point, index of refraction, polarizability, surface tension and molar volume. We can correlate these properties with topological index whose values are displayed in Table 4. In Table 3, we write all values of properties of drugs.

**Table 3:** Properties of drugs.

Sr. no	Drugs	B.P (°C)	D (g/cm^3^)	E.O.V (kJ/MOL)	F.P (°C)	I.O.R	M.R (cm^3^)	P (cm^3^)	S.T (dyne/cm)	M.V (cm^3^)
1	Marbofloxacin	576.9	1.6	90.9	302.7	1.711	85.5	33.9	81.5	218.5
2	1-Octacosanol	428.1	0.8	78.9	135.3	1.458	133.3	52.8	32.2	488.2
3	L-threonic acid	518.9	1.7	91.1	281.7	1.56	26.7	10.6	95.9	82.5
4	Menthyl Salicylate	362	1.1	63.2	140.4	1.533	78.9	31.3	42	254.3
5	Florfenicol	617.5	1.5	96.3	327.3	1.548	78.4	31.1	49.1	246.7
6	Emylcamate	233.3	1.0	47	92.7	1.439	39.8	15.8	31.9	151.3
7	Fluindione	406.3	1.3	65.8	154.5	1.619	63.1	25	51.5	179.9
8	Levalbuterol	433.5	1.2	72.7	159.5	1.566	67.8	26.9	49.2	207.6
9	Arbutin	561.6	1.6	88.8	293.4	1.65	63.8	25.3	73.5	174.9
10	Amiloxate	362.8	1	60.9	151.6	1.524	73.7	29.2	35.5	240.8
11	Fluconazole	579.8	1.5	91.2	304.4	1.663	76.1	30.2	55.4	205.3
12	Terbinafine	417.9	1	67.1	183.7	1.586	97.1	38.5	41.1	289.1
13	Clotrimazole	482.3	1.1	71.9	245.5	1.617	105.9	42	44.5	302.8
14	Miconazole	555.1	1.4	80.5	289.5	1.625	104.7	41.5	46.6	296
15	Voriconazole	508.6	1.4	82	261.4	1.617	85.6	33.9	45.2	244.7
16	Tavaborole	230.8	1.3	49.4	93.4	1.526	36.2	14.3	37.2	117.9
17	Butenafine	426.1	1	68.1	187.7	1.598	104.9	41.6	40	307.5
18	Econazole	533.8	1.3	77.9	276.6	1.615	100.1	39.7	45.5	286.7
19	Oxiconazole	576.8	1.4	83.2	302.6	1.635	107.7	42.7	48.4	300.7
20	Sertaconazole	614.1	1.4	87.8	325.2	1.675	114.5	45.4	51.5	304.8

**Table 4:** Topological indices of drugs.

Sr. No	Drug	$NM_1$	$NM_2$	NABC	$NF_1$	$ND_1$	$ND_2$	$ND_3$	$ND_4$	$ND_5$
1	Marbofloxacin	342	1081	15.3037	2274	168.1315	8.22	14802	5.1066	62.4496
2	1-Octacosanol	216	420	17.4021	844	107.8272	10.1356	3300	7.3938	56.5
3	L-threonic acid	76	178	4.8365	402	36.6954	2.6472	1866	1.8513	18.6
4	Menthyl Salicylate	230	639	11.7726	1350	113.2778	6.4512	7600	4.0803	44.8783
5	Florfenicol	230	631	11.8373	1352	112.7555	6.4489	7420	4.1131	45.9357
6	Emylcamate	88	219	5.403	484	42.6679	2.9595	2450	2.0687	20.8381
7	Fluindione	238	728	10.8886	1534	117.2421	5.9196	9636	3.6164	42.7952
8	Levalbuterol	180	480	9.6343	1006	88.7696	5.2968	5394	3.4013	36.3143
9	Arbutin	224	632	11.1582	1334	110.1373	6.0697	7580	3.8215	43.5119
10	Amiloxate	170	406	10.5498	834	84.3311	5.9227	4016	3.9769	37.4167
11	Fluconazole	270	780	13.2539	1628	133.5172	7.2838	9660	4.5235	50.2528
12	Terbinafine	221	595	11.8403	1231	109.5515	6.5666	6792	4.1795	43.4774
13	Clotrimazole	328	1039	15.4504	2232	161.2871	8.5014	15288	5.3434	59.3889
14	Miconazole	291	790	15.0764	1631	144.191	8.3012	8922	5.1972	56.2952
15	Voriconazole	338	1052	15.2759	2282	165.4146	8.2751	14662	5.0785	61.1079
16	Tavaborole	136	389	6.6011	812	67.203	3.6074	4690	2.2185	25.2976
17	Butenafine	290	816	14.3448	1702	143.4648	7.8754	9658	4.8593	54.2607
18	Econazole	278	752	14.5368	1548	137.9291	8.0295	8482	5.0375	53.7976
19	Oxiconazole	302	823	15.659	1708	149.4745	8.6212	9412	5.4143	58.5833
20	Sertaconazole	340	978	16.4861	2036	168.1814	9.0318	11890	5.5543	62.844

Given below table is for showcase of results of each model get after performing some statistical analysis. To find which index has higher capacity of prediction, we set a parameter that index $R^2$ value between 0.9 or greater then 0.99 fall in category of best predictor. Below Table 5 are formedd with the help of above mentioned parameter.

**Table 5:** Combined outcomes of linear, quadratic and cubic regression models.

Property	Predictors	Linear $R^2$	Quadratic $R^2$	Cubic $R^2$	Best Predictor
M.R	NM1	0.555	0.614	0.618	ND4
	NM2	0.352	0.433	0.438	
	NABC	0.880	0.882	0.892	
	NF1	0.323	0.406	0.413	
	ND1	0.567	0.620	0.624	
	ND2	0.905	0.907	0.911	
	ND3	0.214	0.271	0.273	
	ND4	0.914	0.921	0.922	
	ND5	0.759	0.768	0.783	
P	NM1	0.550	0.61	0.618	ND4
	NM2	0.350	0.43	0.439	
	NABC	0.870	0.88	0.892	
	NF1	0.323	0.407	0.414	
	ND1	0.560	0.62	0.625	
	ND2	0.902	0.907	0.911	
	ND3	0.214	0.272	0.273	
	ND4	0.913	0.920	0.922	
	ND5	0.759	0.768	0.784	
M.V	NM1	0.277	0.382	0.382	ND4
	NM2	0.121	0.207	0.239	
	NABC	0.679	0.697	0.777	
	NF1	0.105	0.181	0.219	
	ND1	0.287	0.386	0.387	
	ND2	0.734	0.768	0.844	
	ND3	0.048	0.073	0.088	
	ND4	0.840	0.861	0.880	
	ND5	0.506	0.524	0.554	

Based on the regression studies reported in Table 5 it can be seen that not every index exhibits strong predictive capacity across all physicochemical properties. Of the properties studied, the most correlated with the neighborhood-based indices were molar refractivity (M.R) and polarizability (P). Specifically, $ND_4$ had high predictive power for both M.R and P in linear, quadratic, and cubic models, with coefficient of determination values up to 0.922 ($ND_4$, cubic model). Following the same order in molar volume (M.V), $ND_4$ were again seen highly predictive and showed good results with the cubic regression model ( $R^2$ = 0.880). Nevertheless, other properties such as the boiling point (B.P), density (D), enthalpy of vaporization (E.O.V), flash point (F.P), index of refraction (I.R), and surface tension (S.T) did not achieve the predefined strong prediction threshold ($R^2$ = 0.7) of most of the indices. Generally, it is clear that the cubic regression model is always more effective than both the linear and quadratic regression models, which means that nonlinear structural influences have a big role to play in these two physicochemical properties, as M.R and P.

## Predictive modeling and quantitative analysis

After finding values of targeted topological indices and properties of drugs now our next step is to find and establish relation between them. For this purpose we take help of different models as linear, quadratic and cubic to find which one explain the relation in a better way and to find best curve which fits in given situation . Mathematically below equations represent these models as :

2 $$\begin{aligned} & P = \alpha + \beta (T) \end{aligned}$$

3 $$\begin{aligned} & P = \alpha + \beta (T) + \gamma (T)^2 \end{aligned}$$

4 $$\begin{aligned} & P = \alpha + \beta (T)^3 + \gamma (T)^2 + \delta (T) \end{aligned}$$

Above equations are general form of our models in which P is the representative of physical attributes, T denotes topological indices and *α*, *β*, *γ*, *δ* play role of coefficients of equations. We perform analysis by making base of data available in Table 5. We use different statistical parameter which prove to be beneficial for finding better results like to show number of observation we show it with N. Similarly we conditioned to take P value less than 0.005 and if we are interested to find intensity of relation is between indices and property we take helps of two coefficients named as correlation coefficient symbolically write as R and coefficient of determination as $R^2$. If values of $R^2$ are near to 1 then it is the symbol of strong connection and vice versa. Keeping these things in mind we generate Table 6 which we devote to linear regression model in which a is coefficient of topological index and b is constant of equation while Table 7 is given to quadratic regression where c is coefficient of quadratic part, d is numerical value of linear and e is the constant in equation. In the last Table 8 slot is occupied by cubic regression models in which f is cubic part constant, g belong to quadratic part and h and i is for linear and constant part of equation.

**Table 6:** Statistical calculation of best predictors of linear regression models.

Regression Equation	N	a	b	p	F	R	$R^{2}$	$S_{res}$
M.R = 19.577($ND_4$) − 3.259	20	19.577	− 3.259	0.001	176.335	0.995	0.914	1177.692
P = 7.757($ND_4$) − 1.275	20	7.757	− 1.275	0.001	175.277	0.955	0.913	186.034
M.V = 60.529($ND_4$) − 17.348	20	60.529	− 17.348	0.001	87.079	0.915	0.840	22846.070

**Table 7:** Statistical calculation of best predictors of quadratic regression models.

Regression Equation	N	c	d	e	p	F	R	$R^{2}$	S_res_
M.R = $-0.908(ND_{4})^{2} + 27.361(ND_{4}) -18.418$	20	− 0.908	27.361	− 18.418	0.001	91.562	0.959	0.921	1076.192
P = $-0.364(ND_{4})^{2} + 10.877( ND_{4}) - 7.350$	20	− 0.364	10.877	10.877	0.001	91.171	0.959	0.920	169.736
M.V = $4.951(ND_{4})^{2} + 18.153(ND_{4}) + 65.299$	20	4.951	18.153	65.299	0.001	48.430	0.926	0.861	19829.016

**Table 8:** Statistical calculation of best predictors of cubic regression models.

Regression Equation	N	f	g	h	i	p	F	R	$R^{2}$	S_res_
M.R = $-0.145(ND_4)^3 +1.099(ND_4)^2 +18.968(ND_4) - 8.107$	20	− 0.145	1.099	18.968	− 8.107	0.001	57.498	0.959	0.922	1071.512
P = $-0.059(ND_4)^3 +0.448(ND_4)^2 +7.481(ND_4) - 3.178$	20	− 0.059	0.448	7.481	− 3.178	0.001	57.263	0.959	0.922	168.970
M.V = $3.443(ND_4)^3 - 42.598(ND_4)^2 +216.946(ND_4) -178.924$	20	3.443	42.598	216.946	− 178.924	0.001	35.652	0.936	0.880	17203.506

### Graphical analysis

**Fig. 4:**
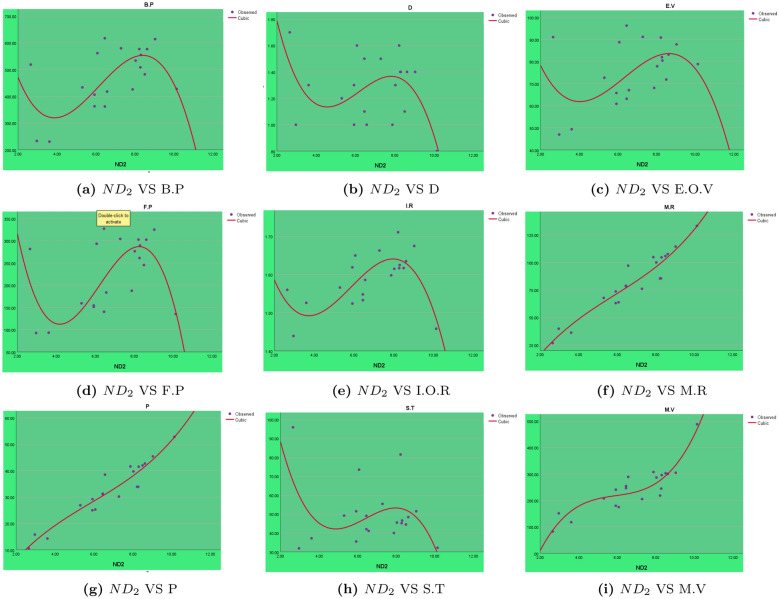
Regression plots corresponding to the index. $ND_2$.

**Fig. 5:**
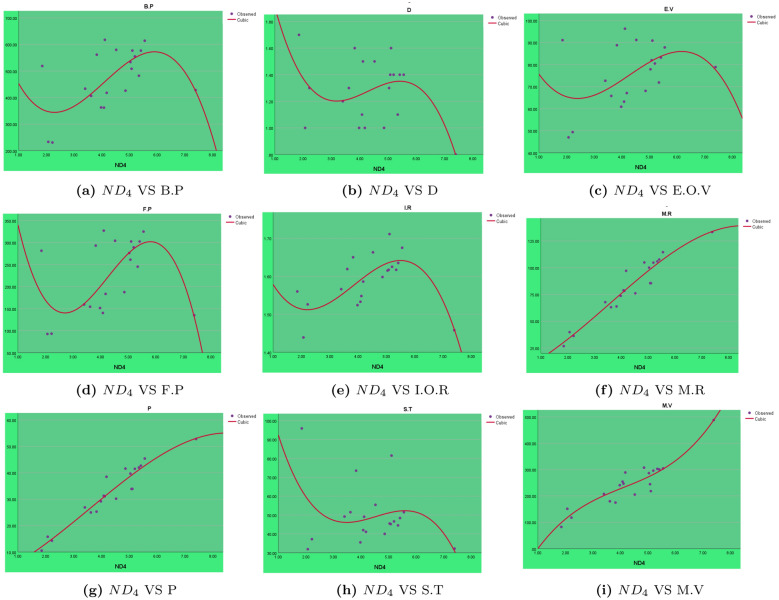
Regression plots corresponding to the index. $ND_4$.

This section is dedicated to showing the methodology we perform above in a pictorial way using graphs. Graphs prove to be a strong tool to demonstrate the intensity of the relationship between indices and properties and also provide a medium of comparison in which we are able to decide which regression model fits the situation and gives better results. We analyze $ND_2$ with properties and show the outcomes of cubic regression analyses in Fig. 4. Likewise, Fig. 5 shows the relationship between $ND_4$ and properties. Collectively, each plot provides an understanding of the predictive ability of each model and how closely they correlate with the provided data.

### Equations

In our study we tested different models and all of these cubic perform best so in this section we try to show out analysis in equation by using general equation of cubic regression model shown in Eq. (4). Here below by taking care of drugs characteristic as dependent variable and topological indices as independent we write equation corresponding to every neighbourhood index. By making cubic regression model as foundation we employ indices to forecast outcomes of short listed properties shown in Table 3.

Using $ND_2$, we developed models to predict the following outcomes: BP, D, E. O. V, F. P, I. O. R, M. R, P, S. T, and M. V.

5 $$\begin{aligned} & B.P= -5.009(ND_2)^3 +92.152(ND_2)^2 -487.915(ND_2) +1118.020 \end{aligned}$$

6 $$\begin{aligned} & D= -0.013(ND_2)^3 +0.249(ND_2)^2 -1.432(ND_2) +3.761 \end{aligned}$$

7 $$\begin{aligned} & E.O.V= -0.443(ND_2)^3 +8.425(ND_2)^2 -46.288(ND_2) + 140.642 \end{aligned}$$

8 $$\begin{aligned} & F.P= -5.164(ND_2)^3 +96.296(ND_2)^2 -534.344(ND_2) + 1040.613 \end{aligned}$$

9 $$\begin{aligned} & I.O.R= -0.004(ND_2)^3 +0.068(ND_2)^2 -0.347(ND_2) +2.038 \end{aligned}$$

10 $$\begin{aligned} & M.R = 0.201(ND_2)^3 -3.537(ND_2)^2 + 31.762(ND_2) -34.764 \end{aligned}$$

11 $$\begin{aligned} & P= 0.078(ND_2)^3 -1.380(ND_2)^2 +12.470(ND_2) -13.583 \end{aligned}$$

12 $$\begin{aligned} & S.T= -0.747(ND_2)^3 +14.366(ND_2)^2 -86.781(ND_2) +210.291 \end{aligned}$$

13 $$\begin{aligned} & M.V= 2.764(ND_2)^3 -49.079(ND_2)^2 +300.899(ND_2) -416.726 \end{aligned}$$

Keeping $ND_4$ as index we build and makes equation of cubic regression as:

14 $$\begin{aligned} & B.P= -9.685(ND_4)^3 +119.054(ND_4)^2 -393.096(ND_4) +736.483 \end{aligned}$$

15 $$\begin{aligned} & D= -0.027(ND_4)^3 + 0.344(ND_4)^2 -1.382(ND_4) +2.971 \end{aligned}$$

16 $$\begin{aligned} & E.O.V= -0.796(ND_4)^3 +10.227(ND_4)^2 -35.312(ND_4) +101.514 \end{aligned}$$

17 $$\begin{aligned} & F.P= -10.837(ND_4)^3 +138.331(ND_4)^2 -510.369(ND_4) +723.337 \end{aligned}$$

18 $$\begin{aligned} & I.O.R = -0.007(ND_4)^3 +0.085(ND_4)^2 -0.268(ND_4) +1.768 \end{aligned}$$

19 $$\begin{aligned} & M.R = -0.297(ND_4)^3 -3.191(ND_4)^2 +10.514(ND_4) -1.783 \end{aligned}$$

20 $$\begin{aligned} & P = -0.119(ND_3)^3 +1.278(ND_3)^2 + 4.12(ND_4) +0.749 \end{aligned}$$

21 $$\begin{aligned} & S.T = -1.328(ND_4)^3 + 18.082(ND_4)^2 -77.651(ND_4) +153.303 \end{aligned}$$

22 $$\begin{aligned} & M.V = 3.388(ND_4)^3 -41.839(ND_4)^2 +213.877(ND_4) -175.333 \end{aligned}$$

## Comparison with existing studies

To critically evaluate the predictive performance of the neighbourhood degree sum indices employed in the present study,a direct comparison is drawn with the findings reported by Tamilarasi and Balamurugan^28^ in which they employed both degree based and neighbourhood degree sum indices in QSPR and QSTR modelling of antifungal drugs, targeting pharmacokinetic properties and toxicity ($LD_{50}$) but limiting their regression analysis to linear models. Their exclusive focus on ADMET (absorption, distribution, metabolism, excretion, and toxicity related properties) endpoints and linear frameworks leaves a gap in understanding nonlinear structural contributions to physicochemical behaviour. The present study complements their work by systematically comparing linear, quadratic, and cubic regression models across nine neighbourhood indices, showing that cubic regression consistently yields the highest predictive accuracy, a finding not addressed in a linear only approach. Similarly, Parveen et al.^18^ studied thirteen fungicides using classical degree based topological indices for physicochemical properties but did not incorporate neighbourhood degree sum indices. The current investigation extends this work by applying neighbourhood based descriptors, which encode richer local structural information through the surrounding atomic environment rather than relying solely on vertex degree. The superior performance of $ND_{4}$ confirms that neighbourhood level information offers added discriminating power for modelling electronic properties such as molar refractivity and polarizability compared to conventional degree based descriptors. Taken together, these comparisons highlight three distinguishing contributions of the present study. First one is a larger and more chemically diverse set of twenty antifungal drugs. Second, systematic evaluation of nine neighbourhood degree sum indices across three regression models, enabling transparent comparison of predictive performance and third is identification of $ND_{4}$ as a consistently strong predictor of molar refractivity and polarizability, providing a clear recommendation for future QSPR studies targeting electronic properties of antifungal compounds. Higher order regression models gave better predictive accuracy, but their added complexity carried a risk of overfitting. Across all regression methods we tested, $ND_4$ consistently stood out as a strong predictor. This consistency suggests that the structure property relationships we observed are not just byproducts of any single modeling technique rather it supports the dependability of our findings. That said, the sample size is relatively small, so the results warrant careful interpretation. To better test how well the models generalize, future studies should include larger datasets and external validation.

## Conclusion

Among the nine neighbourhood degree sum indices tested, $ND_4$ (Fourth neighbourhood degree product) showed the highest predictive capacity. For molar refractivity and polarizability, the cubic regression model using $ND_4$ achieved $R^2 = 0.922$ in both cases, and for molar volume $R^2 = 0.880$. This indicates that neighbourhood indices capture second order structural effects which correlate strongly with electronic and refractivity related properties. In contrast, properties such as boiling point, density, flash point, and surface tension yielded poor correlations ($R^2 < 0.7$) across all indices, suggesting that these are dominated by intermolecular forces not encoded in neighbourhood degree sums. The cubic model consistently outperformed linear and quadratic models, but the improvement over quadratic was marginal (e.g., $R^2 = 0.921$ for M.R).

In summary, neighbourhood topological indices especially $ND_4$ are effective for modelling molar refractivity and polarizability of antifungal drugs, but their utility is limited for properties governed by long range or bulk intermolecular interactions.

## Future directions

The topological indices of the present analysis are limited to the degree sum, and it is a possible direction of the work to extend the analysis further. Although these indices are useful structural indicators, and have been found to be predictive, other classes of indices like distance-based, degree distance, spectral and information-theoretic indices might have higher fidelity to chemical graph features which are not well represented by degree sum measures. Thus, the subsequent research can utilize such alternative indices or a combination of them in order to come up with more robust and effective regression/QSPR models of antifungal drugs molecules. The expanded approach would also increase predictive efficiency and conceptualize more about property relationships within the structure.

## Data Availability

The datasets used and/or analysed during the current study are available from the corresponding author on reasonable request.
